# Spontaneous Pneumomediastinum, Pneumothorax, Pneumopericardium and Subcutaneous Emphysema—Not So Uncommon Complications in Patients with COVID-19 Pulmonary Infection—A Series of Cases

**DOI:** 10.3390/jcm10071346

**Published:** 2021-03-24

**Authors:** Talida Georgiana Cut, Cristina Tudoran, Voichita Elena Lazureanu, Adelina Raluca Marinescu, Raluca Dumache, Mariana Tudoran

**Affiliations:** 1 Department XIII, Discipline of Infectious Diseases, University of Medicine and Pharmacy “Victor Babes” Timisoara, E. Murgu Square, Nr. 2, 300041 Timisoara, Romania; Talida.cut@gmail.com (T.G.C.); vlazureanu@gmail.com (V.E.L.); marinescu.adelina@umft.ro (A.R.M.); 2 Department VII, Internal Medicine II, Discipline of Cardiology, University of Medicine and Pharmacy “Victor Babes” Timisoara, E. Murgu Square, Nr. 2, 300041 Timisoara, Romania; tudoran.mariana@umft.ro; 3 County Emergency Hospital “ Pius Brinzeu”, L. Rebreanu Str., Nr. 156, 300041 Timisoara, Romania; 4 Center of Molecular Research in Nephrology and Vascular Disease, Faculty of Medicine, University of Medicine and Pharmacy “Victor Babes” Timisoara, E. Murgu Square, Nr. 2, 300041 Timisoara, Romania; 5 Department VIII, Discipline of Forensic Medicine, University of Medicine and Pharmacy “Victor Babes” Timisoara, E. Murgu Square, Nr. 2, 300041 Timisoara, Romania; raluca.dumache@umft.ro

**Keywords:** COVID-19, spontaneous pneumomediastinum, pneumopericardium, pneumothorax, subcutaneous emphysema, inflammation, cytokine storm

## Abstract

(1) Background: Spontaneous pneumomediastinum (PM), pneumothorax (PT), and pneumopericardium (PP) were recently reported as rare complications in patients with severe COVID-19 pneumonia, and our study aims to follow the evolution of these involvements in 11 cases. The presumed pathophysiological mechanism is air leak due to extensive diffuse alveolar damage followed by alveolar rupture. (2) Methods: We followed the occurrence of PM, PN, PP, and subcutaneous emphysema (SE) in 1648 patients hospitalized during the second outbreak of COVID-19 (October 2020–January 2021) in the main hospital of infectious diseases of our county and recorded their demographic data, laboratory investigations and clinical evolution. (3) Results: Eleven patients (0.66%) developed PM, with eight of them having associated PT, one PP, and seven SE, in the absence of mechanical ventilation. Eight patients (72.72%) died and only three (27.27%) survived. All subjects were nonsmokers, without known pulmonary pathology or risk factors for such complications. (4) Conclusions: pneumomediastinum, pneumothorax, and pneumopericardium are not so uncommon complications of SARS-CoV2 pneumonia, being observed mostly in male patients with severe forms and associated with prolonged hospitalization and poor prognosis. In some cases, with mild forms and reduced pulmonary injury, the outcome is favorable, not requiring surgical procedures, mechanical ventilation, or intensive care stay.

## 1. Introduction

COVID-19 has become the largest pandemic in recent centuries and is associated with increased morbidity and mortality, as well as a large spectrum of complications. Several articles, published in the medical literature worldwide [1,2,3,4], debate sporadic cases of spontaneous pneumomediastinum (PM), pneumothorax (PT), pneumopericardium (PP), and subcutaneous emphysema (SE) diagnosed in patients with SARS-CoV2 pneumonia, even in the absence of mechanical ventilation associated with barotrauma. Subsequently, several retrospective studies [4,5], the largest one being a multicenter analysis of Martinelli et al. [3], focused on this topic. The principal pathophysiologic mechanism of PM is represented by the Macklin phenomenon, explaining the development of an increased pressure gradient between the marginal alveoli and the lung parenchyma, which, in the presence of the extensive alveolar injury, determines air leakage along the surrounding bronchovascular sheaths into the mediastinum. It is known that inflammation could render the alveolar wall more prone to rupture, which could be exacerbated by a persistent cough or any factors increasing the intra-alveolar pressure.

Some studies included both patients who developed PT and/or PM spontaneously, as well as those occurring during invasive positive pressure ventilation [3], where barotrauma could represent the responsible mechanism. In our study, we focused only on patients who developed these complications spontaneously during the course of COVID-19, with some of them having initially mild/moderate forms of the disease that worsened gradually [6].

The aim of this study was to follow the characteristics of patients hospitalized for COVID-19 who developed PT, PM, PP, and SE and to observe which factors seem to influence the evolution and prognosis of these complications.

## 2. Materials and Methods

### 2.1. Study Population

This is a retrospective case series, describing the evolution of 11 patients out of all 1648 cases hospitalized in the clinics of the Hospital for Infectious Diseases during the second outbreak of COVID-19 from 1 October until 31 January.

### 2.2. Methods

We recorded their demographic data, history, clinical characteristics, laboratory data, and thorax computer tomography (CT) results and therapy and followed their evolution and outcome. All patients were able to sign and signed, at admission to the hospital, the standardized informed consent form required by the national health system of our country, by which they consented to their data being used for research and medical education purposes. The study was approved by the Ethics Committee of the hospital Nr. 2035.

### 2.3. Statistical Methods

The Statistical Package for the Social Sciences v.25 (SPSS, Chicago, IL, USA) was employed to perform data analysis. We presented continuous variables as mean and standard deviation (SD) or median and interquartile range (IQR) and categorical variables as frequency and percentages. We considered *p*-values under 0.05 to indicate statistically significant differences.

## 3. Results

In this study, we followed the evolution of 11 patients with COVID-19, nine men and two women, aged between 36 and 78 years, mean age 58.27 ± 12.39 years, hospitalized after two to seven days, median 4.8 (2–7) days, since the onset of symptoms of SARS-CoV-2 infection, the diagnosis being confirmed by a real-time polymerase chain reaction (PCR). On arrival, three patients presented a medical history of hypertension, two of type 2 diabetes mellitus, and three of them obesity. All patients denied alcohol consumption and tobacco or recreational drug use. After 1 to 15 days of hospitalization, with a median of 4.45 (2–6) days, all subjects developed at least one of the following complications: PT, PM, PP, and SE. Five patients presented low oxygen saturation on room air, with values ranging between 50% and 86%, which improved up to 90–93% on a non-rebreather mask at 15 L/min, Table 1.

**Figure 1:**
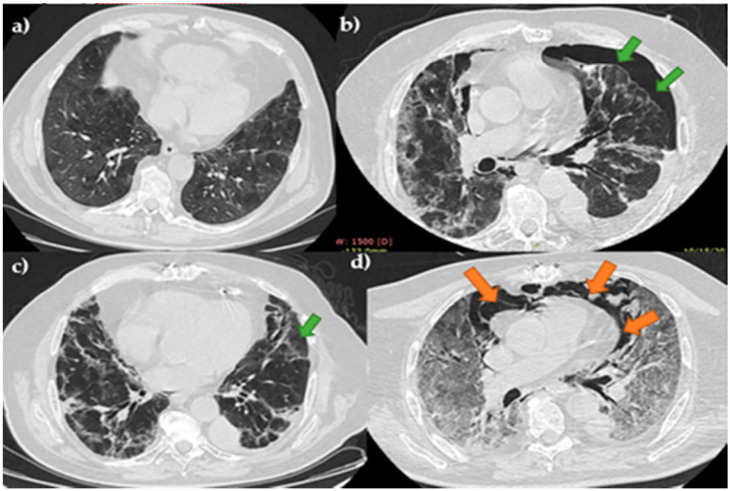
Case 1–patient admitted with moderate COVID-19 injury, developed PT and subsequently PM. **Legend:** PT-pneumothorax—green arrow; PM-pneumomediastinum—orange arrow; (**a**) initial CT with moderate injury; (**b**) right PT; (**c**) control CT after surgical drainage of PT; (**d**) PM and severe pulmonary injury.

**Figure 2:**
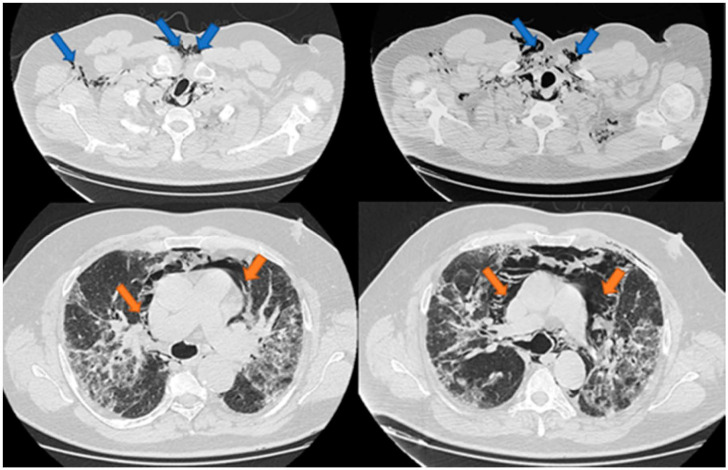
Case 2-Pneumomediastinum and subcutaneous emphysema. Legend: PM—pneumomediastinum—orange arrow; SE—subcutaneous emphysema—blue arrow.

**Figure 3:**
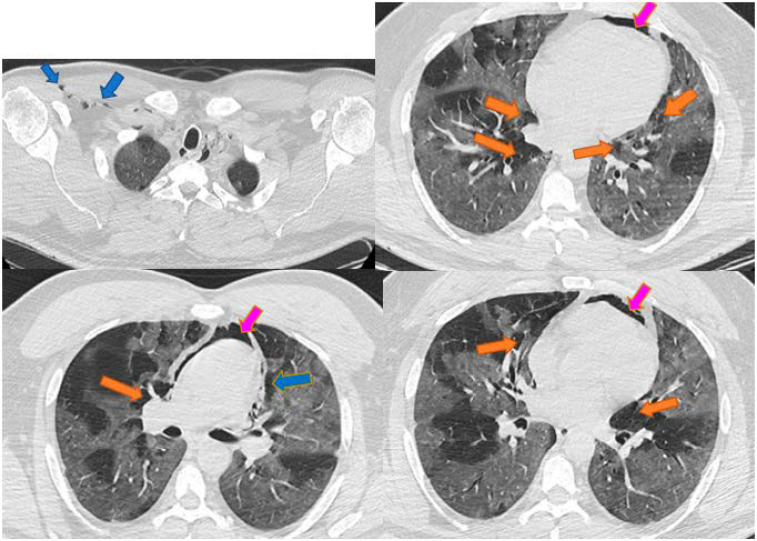
Case 3: Pneumomediastinum, pneumopericardium, and subcutaneous emphysema. Legend: PM—pneumomediastinum—orange arrow; PP—pneumopericardium—magenta arrow; SE—subcutaneous emphysema—blue arrow.

An initial thorax CT was performed in order to assess the presence and severity of the pulmonary injury caused by SARS-CoV-2 infection. Among the most characteristic radiologic aspects were ground-glass opacities, consolidated opacities, and septa thickening. Four patients had, at admission mild pulmonary injury (10–35%), three moderate lesions (50%) and four had severe forms (over 70%). In none of the cases were PT, PM, PP, or SE present at the presentation in the emergency room, but they occurred after 1 to 15 days of hospitalization, median 4.45 (2–6) days, being associated usually with the aggravation of the pulmonary injury. Patients were monitored, in terms of vital signs, electrocardiogram, oximetry, arterial blood gases, and biological parameters to adjust therapy. Thorax CT images of 3 patients with such complications are presented in Figure 1, Figure 2 and Figure 3.

Laboratory studies evidenced elevated white cell count with neutrophilcount, median 90 (84.4–95.3)., and decreased lymphocyte count, median 5.4 (2.4–7.7). C reactive protein (CRP) presented values above 100 mg/L in seven patients, median 113.75 (53.04–247). Elevated levels of ferritin (median 2132.66 (808–2692.1)), fibrinogen (median 8.45 (5.5–9.87)), and interleukine (Il)-6 (median 87.01 (29–1245)) were observed in all patients. PH values varied during the evolution, from a median of 7.4 (7.34–7.46) initially to 7.37(7.3–7.42, *p* ˂ 0.001) finally, and lactate had an ascendant tendency from a median of 30.9 (19.63–43.28) initially to 32.2 (19.5–45.67, *p* ˂ 0.001) finally. Arterial blood gases were as follows: PO_2_ initially median 78 (59–83) and in evolution 54 (48–85) mmHg, with *p* ˂ 0.001; PCO_2_ initially median = 50 (46–58) and in evolution 44.3 (42–69) mmHg, *p* ˂ 0.001; and O_2_ saturation from a median of 93 (90–94.3) initially to 69.5 (65.4–96), *p* ˂ 0.001 finally. In evolution, six patients were associated with elevated procalcitonin levels, median 1.21 (0.15–5.87), which is highly suggestive of sepsis, Table 2.

All patients completed a course of ceftriaxone associated with levofloxacin, dexamethasone sodium phosphate, and nadroparin. Remdesivir, tocilizumab, and anakinra were administrated to six patients (with age between 36 and 61 years, with severe pulmonary lesions and elevated Il-6 and Il-1). Throughout hospitalization, 10 patients needed supplemental oxygen supply and underwent frequent self-proning. Eight patients had to be transferred to the intensive care unit (ICU), and invasive ventilation was performed. One patient required additional extracorporeal membrane oxygenation (ECMO) because he maintained hypoxic respiratory failure despite optimization of mechanical ventilation. Cardiothoracic surgery specialists were consulted in all cases, and five patients required intercostal chest drain insertion while the rest were managed conservatively.

Eight patients died after 12 to 40 days, median 23.5 (14–33.75) days of hospitalization, with a median interval of 19.5 (9.25–25.5) days after the occurrence of the air leak. The remaining three, with reduced pulmonary injury at admission, did not require surgical drainage and were discharged in good clinical condition after a median in-hospital stay of 7 (2–7) days.

## 4. Discussion

As the COVID-19 pandemic evolved and the number of cases increased worldwide, several scientific papers were published in the medical literature, starting with March 2020 [7,8,9,10], reporting patients who had developed spontaneous PT, PM, or even PP, in the absence of invasive mechanical ventilation. These conditions were initially considered rare complications of the SARS-Cov2 pulmonary infection. A literature review by Elhakim et al. [4] analyzed all 15 cases published until June 2020 and concluded that most of the patients had a favorable clinical course; thus, the mortality rate was about 26%. The largest study on this topic, published at the end of August, is that of Martinelli et al., who analyzed the database of COVID-19-treating hospitals in the UK, and described the characteristics of 71 patients with PT, PM, and SE occurring both spontaneously and after mechanical ventilation [3]. By analyzing the database of the two clinics of the Hospital for Infectious Diseases from Timisoara, among all patients hospitalized for SARS-CoV2 infection during the first COVID-19 outbreak (28 February to 31 July 2020), when hospitalization was mandatary for all individuals infected with SARS-CoV 2, in contrast with the cited studies, we found no mention of such complications. On the contrary, after the second outbreak of the pandemic, since 1 October 2020 until the end of January 2021, of all 1648 patients admitted in the hospital, we observed the occurrence of these complications in 11 subjects, leading to a prevalence of 0.66%, similar to around 1% reported for hospitalized patients in the medical literature [3,11,12,13]. As in the other studies, the male gender prevailed (72.72%), and the supposed pathophysiological mechanisms were air leakage through the alveolar walls, damage by inflammation, and damage by the subsequent cytokine storm [1,2,5,14]. In our patients, all these complications occurred spontaneously, after several days of evolution, often coinciding with the aggravation of pulmonary lesions, but in the absence of invasive mechanical ventilation or non-invasive positive pressure ventilation. PT was diagnosed the most frequently (in eight cases—72.72%), followed by PM, associated with SE in all these patients (seven cases—63.63%), while PP was identified only in one subject (9.09%). In contrast to other studies where lower mortality was reported [4,15,16], in our patients, PT, PM, and PP frequently led to a fatal outcome (72.72%), despite intensive care measures, including ECMO in one case. Mortality for ECMO-supported patients with COVID-19 has been associated in the literature with risk factors such as age > 65 years, poor baseline functional status such as severe chronic obstructive pulmonary disease on home O_2_ therapy, pre-ECMO cardiac arrest, or acute kidney injury. Our patient had no absolute contraindications for veno-venous ECMO. The oxygenation and ventilation improved in the first 3 days after ECMO initiation. Nevertheless, it is unclear what the outcome of the patient would have been if he had not received ECMO. In the cases with fatal outcome, clinical status worsening coincided with the augmentation of inflammation markers as procalcitonin, ferritin, fibrinogen, interleukin-6, as well as with deterioration of blood gases, lactate, and PH. In our study, the patients who survived had a less severe pulmonary injury (under 35%) and were hospitalized sooner after the onset of symptoms, with an earlier start of specific therapy to avoid the progression of the existing pulmonary lesions and without secondary microbial infection, expressed by low levels of serum procalcitonin.

## 5. Conclusions

Pneumomediastinum, pneumothorax, and pneumopericardium are not rare complications and are diagnosed more frequently in male patients with severe COVID-19 pneumonia, being associated with prolonged hospitalization and poor prognosis.

## Figures and Tables

**Table 1:** Characteristics of the patients group.

Nr.	Gender	Age	CT Injury	Days until		PT	PM	PP	SE	HR	Sat O_2_	Days of NIV	Days of MV	Days of ECMO	Outcome
				Worse-Ning	Discharge/Death										
1.	M	64	50%	15	36	yes	yes	no	yes	66	86%	2	11	0	fatal
2.	M	61	50%	1	17	no	yes	no	yes	82	67%	0	7	4	fatal
3.	M	49	70%	3	21	no	yes	yes	yes	100	80%	11	4	0	fatal
4.	M	57	70%	6	13	yes	yes	no	yes	95	60%	7	6	0	fatal
5.	F	72	20%	1	2	yes	yes	no	yes	70	92%	0	0	0	good
6.	M	50	10%	2	7	yes	no	no	no	70	98%	0	0	0	good
7.	M	54	50%	5	40	yes	yes	no	yes	120	60%	8	26	0	fatal
8.	M	78	30%	3	26	yes	no	no	no	100	87%	0	2	0	fatal
9.	M	71	35%	4	9	yes	no	no	no	75	90%	0	0	0	good
10.	M	49	80%	3	27	yes	no	no	no	100	93%	2	20	0	fatal
11.	F	36	70%	6	12	no	yes	no	yes	91	50%	0	2	0	fatal

Legend: Nr.—number; CT—thorax computer-tomography; PT—pneumothorax; PM—pneumomediastinum; PP—pneumopericardum; SE—subcutaneous emphysema; HR—heart rate; Sat O_2_—oxigen saturation; NIV—non-invasive ventilation; MV—mechanical ventilation; ECMO—extracorporeal membrane oxygenation.

**Table 2:** Laboratory data of the cases.

	Case 1	Case 2	Case 3	Case 4	Case 5	Case 6	Case 7	Case 8	Case 9	Case 10	Case 11
WBC(/mm^3^)	21,470	29,460	21,750	25,430	8320	5540	43,550	25,430	15,090	13,490	2340
Neutrophyls% Lymphocytes%	86.3	89.1	95.3	96.4	84.4	62.2	90.9	90.7	93.2	95.6	72.2
	7.7	2.4	2.9	1.3	7.3	30.1	5.6	5.4	5.4	1.6	20.1
Ferritin (ug/L)	2132.66	2692.1	2619.3	3457.2	808	172.11	4815.3	2543	1077.1	1206.79	220.03
Fibrinogen (g/L)	6.79	9.87	8.88	˃10	5.5	3.03	8.45	8.99	5.5	˃10	5.2
Il-6 (pg/mL)	43.61	61.31	29.03	1245	87.01	1.5	5000	178.32	10.11	1295	89.36
CRP (mg/L)	113.75	31.67	171.1	245.17	88.22	1.26	163.13	245	53.04	345.91	74.92
Procalcitonin	1.21	0.19	25.69	9.7	0.18	0.02	3.05	5.87	0.15	3.2	0.11
(ng/mL)											

Legend: WBC—white blood cell; N—neutrophils; L-lymphocytes; Il—interleukine; CRP—C reactive protein.

## Data Availability

All available data are presented in the two tables included in the manuscript, therefore if you consider necessary to upload them supplimentary on-line please inform us.
